# Thyroid Dysfunction in Non-Interferon Treated Hepatitis C Patients Residing in Hepatitis Endemic Area

**DOI:** 10.1155/2017/2390812

**Published:** 2017-05-30

**Authors:** Nayab Batool, Shan Elahi, Nazish Saleem, Abrar Ashraf

**Affiliations:** ^1^Institute of Chemistry, New Campus, University of the Punjab, Lahore, Pakistan; ^2^Centre for Nuclear Medicine (CENUM), P.O. Box No. 53, Mayo Hospital, Lahore, Pakistan

## Abstract

**Background:**

Association of thyroid dysfunction (TD) with interferon treatment of HCV is well known to clinicians. However, a few studies have highlighted the role of hepatitis C virus per se in the development of TD. The aim of this study was to know the prevalence of TD in non-interferon treated HCV infected patients referred for thyroid function testing.

**Patients and Methods:**

Among 557 ELISA-positive HCV patients 446 (341 females, 105 males) were selected for this study. Serums FT_4_, FT_3_, and TSH were determined by radioimmunoassay method.

**Results:**

TD was detected in 15.2% of patients: 9.0% hypothyroidism and 6.3% hyperthyroidism. In increasing order subclinical hypothyroidism, overt hypothyroidism, overt hyperthyroidism, and subclinical hyperthyroidism were found in 4.7%, 4.3%, 3.6%, and 2.7% patients, respectively. Overall TD was more common in female than in male HCV patients but the difference was not significant (16.1% versus 12.4%; *p* = 0.648). Hyperthyroidism and subclinical hypothyroidism were slightly more common in female and overall hypothyroidism and overt hypothyroidism in male patients but the difference was not statistically significant (*p* > 0.05). The incidence of TD was relatively high in patients above 36 years (median age) but the difference was not statistically significant either collectively or in gender base groups (*p* > 0.05).

**Conclusion:**

Prior to interferon treatment, HCV infection itself causes biochemical thyroid dysfunction in 15.2% of local HCV patients.

## 1. Introduction

Hepatitis C, caused by hepatitis C virus (HCV), is a global health problem. According to the World Health Organization 3 to 4 million people are newly infected with HCV every year and there are about 180 million patients infected with HCV in the world [1]. In addition to hepatic complications, chronic HCV infection has been suggested to cause extrahepatic disorders [2]. These include hematologic diseases, lymphoproliferative disorders, renal disease, and endocrine diseases. The most frequent and clinically important endocrine diseases are autoimmune thyroid disorders [1–3], reported to develop in 5%–10% of pre- and postinterferon alpha (IFN-*α*) treated HCV patients [4, 5]. A significant number of chronic hepatitis C patients have been reported to develop biochemical thyroid dysfunction (TD; hyperthyroidism and hypothyroidism) before IFN-*α* treatment [6–8]. The potential mechanism proposed for this phenomenon is either direct effect of HCV on thyroid cells, triggering of thyroid autoimmunity by altering immune responsiveness, or both [9]. Recently it is reported that HCV can infect human thyroid cell in vitro [10] and has been detected in thyroid tissue from patients with chronic HCV infection [11].

Pakistan is second after Egypt where prevalence of hepatitis C is alarmingly high. Currently about 10 million people in Pakistan are infected with HCV infection [4]. A large proportion of patients with HCV infection have received interferon injections for its treatment. Thyroid dysfunction after interferon treatment of hepatitis C patients is reported in a number of local studies [4, 12, 13] but only a few studies have determined their prevalence before starting the interferon therapy [14, 15]. Recently we have found that one-fourth of local untreated HCV patients are TPO-Ab positive and are at greater risk of developing thyroid disorders during and after interferon treatment [16]. Keeping these facts in view this study is planned to know prevalence of TD in local HCV patients before interferon therapy.

## 2. Patients and Methods

History and laboratory records of all HCV ELISA-positive patients referred for thyroid testing to CENUM, Mayo Hospital, during January 2013 to December 2014 were reviewed. Among them such patients who were already diagnosed for thyroid diseases and taking thyroid medications or had thyroid surgery were excluded. Similarly HCV patients previously treated with interferon were also excluded. We selected those patients whose serum samples were analyzed for both FT_4_ and TSH concentrations with or without FT_3_ determination. In these patients serums FT_4_ and FT_3_ were determined by radioimmunoassay (RIA) and TSH was estimated by IRMA techniques using commercial kits of Immunotech Inc. (Beckman, Czech Republic). RIA and IRMA batches were run with commercially derived control sera at low, medium, and high concentrations which were included in every run. All assays were carried out in duplicate. Measurement of radioactivity, fitting of the standard curve, and analysis of samples were carried out using a computerized gamma counter (Cap-RIA 16, CAPINTEC Inc., USA). RIA and IRMA results were expressed at less than 10% CV of imprecision profile.

Normal ranges for FT_4_, FT_3_, and TSH, as standardized in our laboratory, are 11.0–22.0 pmol/L, 2.5–5.8 pmol/L, and 0.3–4.0 mIU/L, respectively. Hyperthyroidism was classified if serum TSH was ≤0.1 mIU/L and FT_4_ > 22.0 pmol/L and as subclinical if serum TSH was ≤0.1 mIU/L and FT_4_ ≤ 22.0 pmol/L. Hypothyroidism was considered as overt if TSH > 4.0 mIU/L and FT_4_ < 11.0 pmol/L and as subclinical if TSH > 4.0 mIU/L and FT_4_ ≥ 11.0 pmol/L. The analysis of data was carried out using Microsoft Excel program on a personal computer. Chi-square and Student *t*-test were applied to test the significance of difference between two arbitrary groups. A *p* value of less than 0.05 was considered significant.

## 3. Results

Among 557 ELISA-positive newly diagnosed HCV patients 446 were selected for this study. The remaining patients were excluded either due to determination of TSH alone (*n* = 107) or due to combination of FT_3_ and TSH (*n* = 4). In selected patients both serums FT_4_ and TSH were determined with or without additional determination of FT_3_. As serum FT_3_ was determined in only 95 (21.3%) patients we excluded this parameter from data analysis. Among selected patients 341 were female and 105 male patients. Their mean (±SD) age was 37.1 ± 11.1 years with age range between 10 and 90 years and was comparable in male and female patients (35.8 ± 11.6 versus 37.5 ± 11.9; *p* = 0.198). Analysis of thyroid function tests showed that 378 (84.7%) patients had serum TSH level within normal range (0.3–4.0 mIU/L) and were euthyroid. Among the rest of the patients with abnormal TSH (*n* = 68; 15.3%), 40 (9.0%) had hypothyroidism and 28 (6.3%) had hyperthyroidism. Further subgrouping of HCV patients having thyroid disorder revealed that, in increasing order, 21 (4.7%) patients had subclinical hypothyroidism, 19 (4.3%) patients had overt hypothyroidism, 16 (3.6%) patients had overt hyperthyroidism, and 12 (2.7%) had subclinical hyperthyroidism.  Table 1 shows the mean (±SD) level of FT_4_ and TSH in euthyroid, hypothyroid, and hyperthyroid HCV patients. Compared to euthyroid patients serum FT_4_ concentration was significantly lower in hypothyroid and higher in hyperthyroid patients (both *p* < 0.001). Likewise serum TSH concentration was significantly higher in hypothyroid and lower in hyperthyroid as compared to euthyroid HCV patients.

The gender base difference in incidence of TD is elaborated in Table 2. Overall TD was more common in female than in male HCV patients but the difference was not significant (16.1% versus 12.4%; *p* = 0.648). Hyperthyroidism (overt and subclinical) and subclinical hypothyroidism was slightly more common in female as compared to male HCV patients. However, in none of them was the difference statistically significant. Only overall hypothyroidism and overt hypothyroidism were more frequent in male HCV patients but the difference was not statistically significant (*p* > 0.05). Thyroid swelling was detected in 19 (5.6%) patients and all of them were females. The incidence of TD was significantly higher in these women as compared to those women presenting without goiter (36.8% versus 14.9%; *p* = 0.044). Seven among them had TD (two hypothyroidism, five hyperthyroidism). However, excluding these goitrous women from analysis slightly reduced the incidences of hypothyroidism (8.8% to 8.7%) as well as hyperthyroidism (7.3% to 6.2%) but statistical difference remained comparable (*p* > 0.05) between female and male HCV positive counterparts.

The age range in our patients was 10–90 years and the median age was 36 years. To elucidate the role of patient age in incidence of TD, patients were divided on the basis of median age.  Table 3 shows the incidence of TD in HCV patients grouped according to median age. The incidence of TD was relatively high in patients above 36 years of age but the difference was not statistically significant (*p* > 0.05) either collectively or in gender base groups.

## 4. Discussion

The aim of this study was to know the prevalence of thyroid dysfunction in interferon-naive HCV patients referred to CENUM, Mayo Hospital, for thyroid function testing. Our results showed that thyroid dysfunction was detected in 15.2% of the HCV patients. This figure is in accordance with studies carried out in other countries reporting thyroid dysfunction in 7% to 15% untreated HCV patients [6–8, 17]. A recent review of such studies reported 10–15% frequency of TD in interferon-free HCV patients [18]. This high incidence of TD is supported by our previous finding of a high incidence of TPO-Ab (26.8%) in such patients [16]. Local studies carried out in other cities of Pakistan have reported incidence of TD ranging from 7% to 22% in HCV patients before interferon treatment [2, 14, 15]. However, our patient selection method was different from these studies. Unlike these investigations that enrolled consecutively all HCV patients we included only those HCV patients suspected of thyroid dysfunction and hence referred to our Centre for thyroid function testing.

The prevalence of TD in general population is around 5% with more incidence of hypothyroidism compared to hyperthyroidism [19, 20]. According to our results, though overall incidence of TD was much higher than general population, pattern of relatively more hypothyroidism (9.0%) than hyperthyroidism (6.3%) was observed in HCV patients. This result is in accordance with other studies in which higher incidence of hypothyroidism as compared to hyperthyroidism is reported in HCV patients [15, 17]. According to a recent meta-analysis HCV patients are three times more prone to hypothyroidism as compared to control subjects [21]. Keeping in view a high incidence of TPO-Ab in local HCV patients it is expected that this hypothyroidism is probably autoimmune in origin [22]. However, conformation is necessary as nonautoimmune hypothyroidism is also reported in HCV infection [23].

Most studies have reported higher incidence of TD in female HCV patients as compared to male patients because of high incidence of thyroid autoimmunity in female patients [24, 25]. In this study female HCV patients have higher incidence of TD as compared to male patients (16.1% versus 12.4%) but difference was not significant. This observation is similar to a local study [2] and is supported by our previous finding of comparable incidence of TPO-Ab in male and female HCV patients [16]. Although a nonsignificant gender difference of TD incidence is also reported by other studies [26, 27], most plausible reason seems to be low number of male patients (less than one-third of female patients) included in this study. Increasing number of male patients might decrease the percentage of TD in them to a significant level. A previous Chinese study had reported more incidence of TD in female patient even if male patients outnumbered the females [28]. A novel observation in this study was the neck swelling (goiter) detected only in female HCV patients. We found no study in literature to compare this finding. Similarly association of goiter with TD in HCV infected female patients is not reported in any study.

We also investigated incidence of TD in HCV patients of different age groups. It was observed that incidence of thyroid dysfunction was relatively high in patients above 36 years of age but was not statistically more as compared to younger patients. This was true for both male and female HCV patients. This finding is supported by other studies [26, 27] that found no increasing trend of TD with increasing age in HCV patients.

Limitations of this study are its cross-sectional nature, comparatively low number of male patients, serum FT_3_ determination in a few patients, and incomplete medical information like liver enzyme and biopsy status. Recently an association between liver dysfunction and thyroid hormone has been elucidated in HCV patients [28–30]. Besides these we have not determined thyroid autoimmunity related parameters like serums Tg-Ab and TPO-Ab and ultrasound echogenicity in our patients.

In conclusion HCV infection, independent of interferon treatment itself causes biochemical thyroid dysfunction in 15.2% of local patients without age and gender consideration. In Pakistan, local studies have reported TD in 20% of HCV patients after IFN-alpha and ribavirin treatment [13, 31]. It may be speculated that this high incidence is because of preexisting TD in these patients. Thus pretreatment screening is recommended for all HCV patients in whom IFN-*α* therapy is being planned. Thyroid disease need not be a contraindication to IFN-*α* therapy; early detection of subclinical or overt thyroid disease may allow uninterrupted continuation of IFN-*α* treatment. However, periodic monitoring of TD patients should be performed during therapy.

## Figures and Tables

**Table 1:** Mean (±SD) level of FT_4_ and TSH in subgroups of HCV patients.

Subgroup	No.	FT_4_ (pmol/L)	TSH (mIU/L)
Euthyroid	378	15.6 ± 1.8	1.7 ± 0.8
Hypothyroid	40	11.5 ± 6.0^a^	19.6 ± 18.5^b^
Hyperthyroid	28	31.4 ± 19.1^b^	0.08 ± 0.07^a^

FT_4_ = free thyroxine, TSH = thyroid stimulating hormone, supersuffix a = significantly lower than euthyroid patients (*p* < 0.001), and supersuffix b = significantly higher than euthyroid patients (*p* < 0.001).

**Table 2:** Distribution TD in male and female HCV infected patients.

TD	Total (*n* = 446)	Female (*n* = 341)	Male (*n* = 105)	*p* value
Hypothyroidism	40 (9.0%)	30 (8.8%)	10 (9.5%)	0.972
Overt	19 (4.3%)	13 (3.8%)	06 (5.7%)	0.710
Subclinical	21 (4.7%)	17 (5.0%)	04 (3.8%)	0.893
Hyperthyroidism	28 (6.3%)	25 (7.3%)	03 (2.9%)	0.254
Overt	16 (3.6%)	14 (4.1%)	02 (1.9%)	0.559
Subclinical	12 (12.7%)	11 (3.2%)	01 (0.9%)	0.460

Overall TD	68 (15.2%)	55 (16.1%)	13 (12.4%)	0.648

**Table 3:** Effect of patient age on incidence of thyroid dysfunction in HCV infected patients.

Age group	TD	*p* value
All patients		
≤36.0 year (*n* = 232)	27 (11.6%)	0.086
>36.0 year (*n* = 214)	41 (19.2%)	
Female		
≤36.0 year (*n* = 175)	22 (12.6%)	0.189
>36.0 year (*n* = 166)	33 (19.9%)	
Male		
≤36.0 year (*n* = 57)	05 (08.8%)	0.458
>36.0 year (*n* = 48)	08 (16.7%)	
